# Ophiopogonin D improves pancreatic islet cell dedifferentiation to treat diabetes by regulating the GRP78/ROS/PDX1 signaling pathway

**DOI:** 10.3389/fphar.2025.1563201

**Published:** 2025-04-29

**Authors:** Haoxiang Sun, Ruixiang Tan, Yongzhi Sun, Yimeng Li, Ying Xie, Cheng Zhang, Jianping Song, Wei Zhu, Jiuyao Zhou, Changsheng Deng, Manxue Mei

**Affiliations:** ^1^ Artemisinin Research Center, Guangzhou University of Chinese Medicine, Guangzhou, China; ^2^ Sci-Tech Industrial Park, Guangzhou University of Chinese Medicine, Guangzhou, China; ^3^ Dermatology Hospital of Southern Medical University, Guangzhou, China; ^4^ The Second Clinical Medical College, Guangzhou University of Chinese Medicine, Guangzhou, China; ^5^ Department of Pharmacology, School of Pharmaceutical Sciences, Guangzhou University of Chinese Medicine, Guangzhou, China

**Keywords:** ophiopogonin D, dedifferentiation, diabetes, GRP78/ROS/PDX1, unfolded protein response

## Abstract

**Introduction:**

The incidence of diabetes is rising annually, significantly impacting public health and imposing a substantial economic burden on society. Ophiopogonin D (Op D) exhibits certain hypoglycemic effects; however, its mechanisms remain unclear.

**Methods:**

β-cell dedifferentiation, distinct from β-cell apoptosis, is a pathogenic mechanism we aim to explore regarding Op D’s regulatory effects. We established an animal model of β-cell dedifferentiation to assess Op D’s impact on glucose tolerance, blood glucose levels, and insulin secretion. We employed immunohistochemistry and immunofluorescence to analyze the expression levels of dedifferentiation-related proteins. Additionally, we created an *in vitro* β-cell dedifferentiation model using INS-1 cells to evaluate Op D’s influence on insulin secretion and dedifferentiation. Transcriptomic analysis was conducted to explore potential mechanisms by which Op D ameliorates dedifferentiation, with further validation via Western blotting and immunofluorescence. Flow cytometry, fluorescence microscopy, and related assays were used to assess Op D’s effects on oxidative stress. Endoplasmic reticulum (ER) tracing agents marked the ER, and laser confocal microscopy examined ER morphology, with ER stress inducers and inhibitors employed to clarify Op D’s mechanisms.

**Results:**

Results indicated that Op D reduced blood glucose levels, improved glucose tolerance, enhanced insulin secretion, mitigated pancreatic atrophy, and increased PDX1 and FOXO1 expression levels. Furthermore, Op D inhibited ER stress, decreased GRP78 expression, reduced NGN3 levels, elevated PDX1, NKX6.1, and MAFA expression, and decreased oxidative stress-related products (ROS, MDA) while increasing SOD and GSH levels.

**Discussion:**

These findings demonstrate that Op D can improve β-cell dedifferentiation by modulating the GRP78/ROS/PDX1 pathway to inhibit ER stress.

## 1 Introduction

Diabetes mellitus (DM) has emerged as a global pandemic of chronic metabolic dysfunction, representing one of the most pressing public health challenges of our time. According to the 11th edition of the International Diabetes Federation (IDF) Atlas, the worldwide prevalence of diabetes reached 537 million cases in 2021, with type 2 diabetes mellitus (T2DM) comprising approximately 90% of all diagnoses (Federation, 2022). This epidemic continues to escalate at an alarming rate, driven largely by modern lifestyle transitions and shifting dietary patterns (GBD, 2021 Diabetes Collaborators, 2023).

Recent studies have highlighted the critical role of β-cell dedifferentiation—a process in which mature insulin-producing cells regress to progenitor-like states—in the decline of functional β-cell mass (Bilekova et al., 2021). This pathological reprogramming directly contributes to insulin deficiency in T2DM. Consequently, pharmacological interventions targeting the molecular pathways underlying β-cell dedifferentiation have gained traction as a novel therapeutic approach. The development of small-molecule compounds capable of preserving β-cell identity and function represents a promising avenue for future diabetes treatment.

Forkhead box protein O1 (FOXO1) plays a pivotal role in pancreatic β-cells by binding to the promoter region of the pancreatic and duodenal homeobox 1 (PDX-1) gene, thereby reducing chromatin accessibility and impairing the assembly of the transcriptional initiation complex. This transcriptional repression suppresses β-cell proliferation, promotes dedifferentiation, and ultimately results in diminished insulin biosynthesis (Lu et al., 2023; Wang et al., 2023). PDX-1 directly binds to the A3/C1 region (e.g., −250 to −195 bp) of the insulin gene promoter, where it recruits transcriptional coactivators (such as p300/CBP) and chromatin remodeling complexes to enhance chromatin accessibility and facilitate insulin gene transcription. Furthermore, PDX-1 regulates the expression of other β-cell critical genes, including glucokinase (GCK) and glucose transporter 2 (GLUT2), thereby maintaining glucose sensing capacity (Nimmakayala et al., 2021; López-Gil et al., 2024). During insulin resistance, the nuclear localization of FOXO1 is enhanced, further suppressing PDX-1 expression and inhibiting β cell proliferation (Siwan et al., 2024). Dedifferentiated β cells exhibit plasticity and can, under certain conditions, redifferentiate into mature β cells (Bensellam et al., 2012; Teo et al., 2018), providing new insights for diabetes treatment.

Under glucose stimulation, the rate of insulin biosynthesis in pancreatic β cells increases, leading to the potential for misfolded proteins due to factors disrupting proper folding, resulting in unfolded protein response (UPR) and subsequently inducing endoplasmic reticulum (ER) stress (Hagenlocher et al., 2022). UPR is a critical feature of mature, differentiated β cells, and excessive stimulation of UPR under ER stress can lead to metabolic dysregulation and functional failure of β cells (Kupsco and Schlenk, 2015). Glucose-Regulated Protein 78 (GRP78) serves as a central regulator of the UPR, maintaining cellular homeostasis by facilitating protein folding and targeting misfolded proteins for degradation (Kopp et al., 2019). Studies have demonstrated that under pathological conditions such as viral infections or hyperglycemia, ER stress markedly upregulates GRP78 expression, thereby activating downstream signaling pathways (Shu et al., 2020; Yong et al., 2021). In type 2 diabetes mellitus (T2DM), failure of adaptive UPR is associated with β cell dedifferentiation and dysfunction, exacerbating hyperglycemia-induced oxidative stress, which in turn contributes to ER stress, resulting in the gradual loss of β cell characteristics (Verfaillie et al., 2012). Studies demonstrate that GRP78 overexpression may enhance FOXO1 stability through activation of the IRE1α/XBP1 signaling pathway, thereby amplifying its inhibitory effect on PDX1. This cascade reaction accelerates β-cell functional failure, establishing a vicious cycle that promotes diabetes progression (Kamagate et al., 2010). Correcting this ER misfolding can reduce corresponding reactive oxygen species (ROS), thereby restoring normal differentiation of β cells.

Ophiopogonin D (Op D) is a steroid saponin monomer derived from the traditional Chinese medicinal herb Ophiopogon japonicus (Thunb.) Ker Gawl. (Chen et al., 2024). Ophiopogon is considered a health-promoting food in China, and modern pharmacological studies have demonstrated that Op D possesses significant antioxidant properties, improves ER stress, inhibits cell apoptosis, and alleviates inflammation (Lee et al., 2018; Nie et al., 2023; Shen et al., 2024). Op D has been shown to improve blood glucose levels and insulin resistance in mice induced by lipotoxicity while also reducing their blood lipid levels (Zhang et al., 2023). However, research on the hypoglycemic effects of Op D is limited. Therefore, this study aims to investigate whether Op D can improve the dedifferentiation of pancreatic β cells, thereby ameliorating hyperglycemia, and to explore its potential mechanisms.

## 2 Methods and materials

### 2.1 Drugs

**FIGURE 1:**
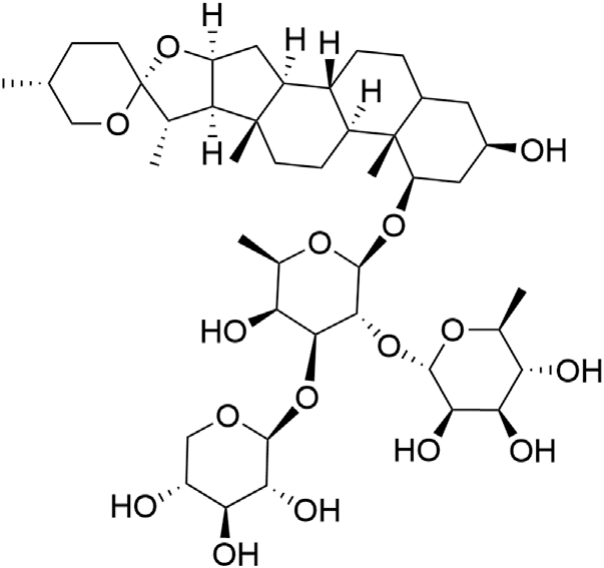
The structure of Op D.

In this study, Ophiopogonin D (Op D) was used as an analytical standard (HPLC purity >98%, structure shown in Figure 1), along with streptozotocin (STZ), and tunicamycin (analytical standard, HPLC purity ≥98%), and tauroursodeoxycholic acid (analytical standard, HPLC purity ≥98%), all purchased from Shanghai Yuanye Bio-Technology Co., Ltd., China. The structural diagram of Op D is illustrated in Figure 1 (constructed using ChemDraw).

### 2.2 Establishment methods of diabetic mouse models and intervention approaches

Sixty male 8-week-old C57BL/6 mice were purchased from the Guangdong Experimental Animal Center (Guangzhou, Guangdong, China) and were housed in a specific pathogen-free (SPF) environment at the Science and Technology Industrial Park of Guangzhou University of Traditional Chinese Medicine. After a week of acclimatization, the mice were randomly divided into six groups: control group (Ctrl), model group (Mm), low-dose Ophiopogonin D group (Op D-low), medium-dose Ophiopogonin D group (Op D -mid), high-dose Ophiopogonin D group (Op D-high), and positive drug glimepiride group (GM), with 10 mice in each group. Except for the Ctrl group, the remaining groups were fed a high-fat, high-sugar diet for 60 days. After 60 days of feeding, the mice were administered an intraperitoneal injection of streptozotocin at a dosage of 50 mg/kg for three consecutive days. Seven days later, fasting blood glucose levels were measured, with a successful model established if fasting blood glucose was ≥11.1 mmol/L.

Following the successful establishment of the model, the Op D-low group received a gavage of 2.5 mg/kgd Ophiopogonin D, the Op D-mid group received 5 mg/kg·d Ophiopogonin D, the Op D-high group received 10 mg/kgd Op D, and the GM group received 5 mg/kg·d, while the Ctrl group received 0.2 mL of 0.9% saline daily. Treatment commenced immediately after model establishment and continued for 30 days, during which the body weight of the mice was measured weekly.

On the 31st day post-treatment, fasting blood glucose levels were measured, followed by a glucose tolerance test on the 32nd day, an insulin tolerance test on the 35th day, and sample collection after a fasting period on the 37th day.

### 2.3 Sample collection and processing

**FIGURE 2:**
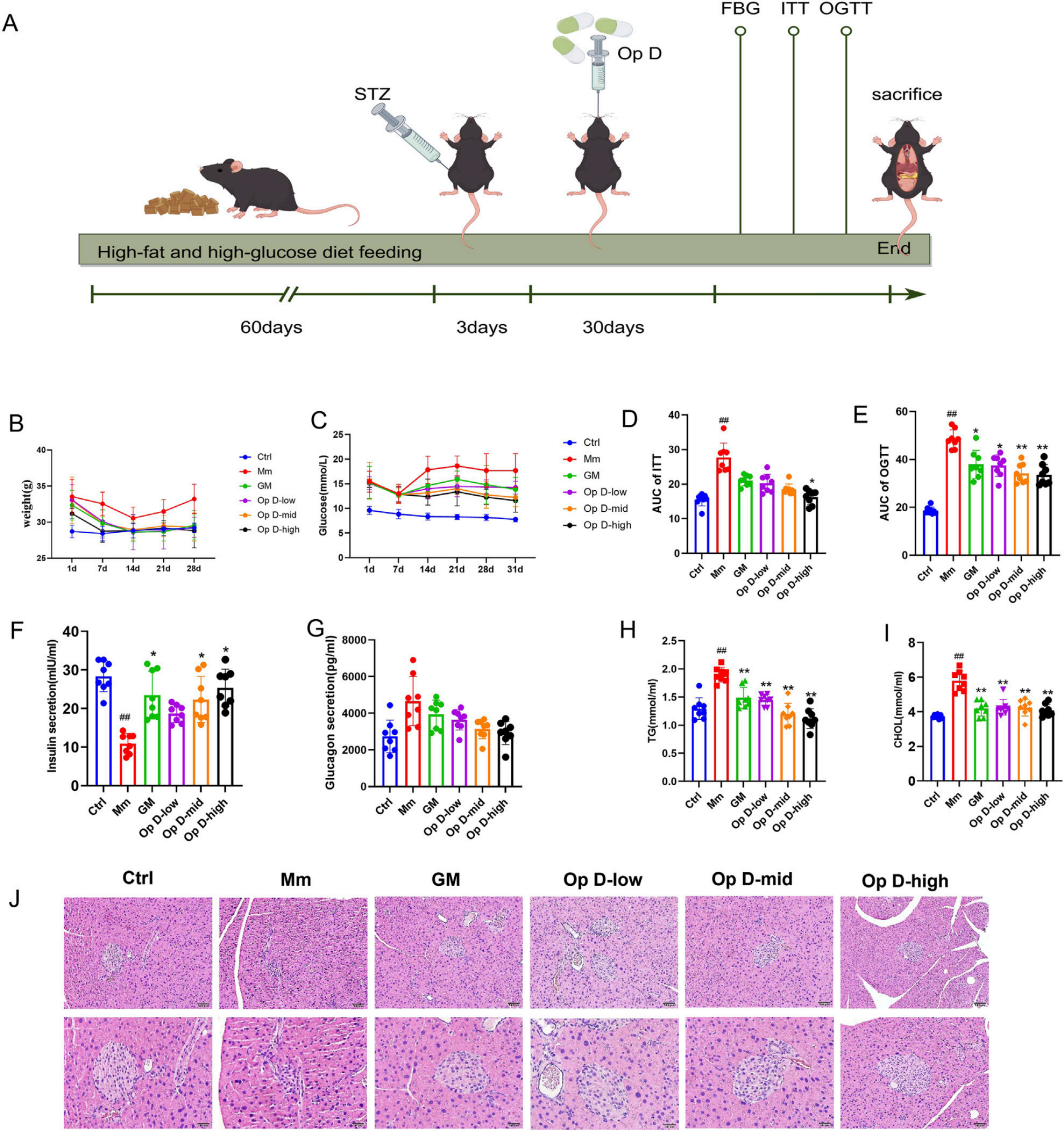
Effects of Op D on Diabetic Mice. **(A)** The time line of *in vivo* experiments. **(B)** Weekly body weight of mice in each group; **(C)** Weekly peripheral blood glucose levels of mice. **(D**,**E)**. OGTT and ITT along with area under the curve statistics; **(F**,**G)** Serum insulin and glucagon levels in mice; **(H**,**I)** Serum TG and CHOL levels in mice; **(J)** HE staining images of mouse pancreas. Scale bar = 50 μm, 20 μm *P < 0.05, **P < 0.01 versus M; ##*P* < 0.01 versus Ctrl.

On day 38 post-administration, mice were anesthetized with pentobarbital sodium (0.3 mL/100 g). Blood samples were collected via cardiac puncture into 1.5 mL Eppendorf tubes. Following blood collection, an abdominal incision was made along the midline using scissors to expose and isolate the pancreas, liver, and spleen. These organs were washed with 0.9% saline and weighed for record-keeping. After weighing, the pancreas was divided into two parts: one was fixed in 4% formaldehyde solution, while the other was stored in a cryogenic tube, which was immediately placed in pre-prepared liquid nitrogen for preservation. The time line of in vivo experiments in Figure 2A.

### 2.4 Hematoxylin-eosin (HE) staining

After euthanizing the mice, pancreatic tissue was collected and subjected to dehydration, embedding, and sectioning for histopathological examination. HE staining was performed using an HE staining reagent (G1121, Solarbio, Beijing, China) following the manufacturer’s instructions.

### 2.5 Immunohistochemistry and immunofluorescence

Paraffin sections were deparaffinized, subjected to antigen retrieval, and treated to block endogenous peroxidase activity. Primary antibodies were incubated overnight at 4°C. The following day, the sections were washed three times with phosphate buffer saline (PBS, Thermo Fisher Scientific, Massachusetts, United States) and incubated with secondary antibodies for 1 h. DAB chromogenic solution was then added to the tissue sections, and color development was observed under a microscope. After rinsing with PBS, the slides were placed in PBS for further processing. Hematoxylin staining was performed to visualize cell nuclei, followed by dehydration and mounting. This procedure was conducted according to the instructions provided with the immunohistochemistry kit (SP-9000, ZSGB-BIO, Beijing, China). For the immunofluorescence assay, fluorophore-conjugated secondary antibodies were used and observed under a fluorescence microscope (TI2-E, Nikon, Tokyo, Janpan), with all other steps following the IHC protocol.

The PDX1 polyclonal antibody (No. 20989-1-AP), FOXO1 polyclonal antibody (Cat No. 18592-1-AP), CoraLite^®^594-conjugated INS polyclonal antibody, and rabbit fluorophore-conjugated secondary antibody were purchased from Proteintech, Wuhan, China.

### 2.6 Detection of serum biochemical parameters in mice

After blood collection, samples were allowed to sit for 4 h, followed by centrifugation at 3,000 rpm for 10 min. The resulting supernatant was collected as serum and sent to the Clinical Testing Center of Guangzhou University of Chinese Medicine for biochemical parameter analysis, including total cholesterol (CHOL) and triglycerides (TG).

### 2.7 Detection of serum insulin and glucagon in mice

Serum obtained from the aforementioned blood samples was analyzed for mouse insulin (P01325) and glucagon (P01275) levels according to the ELISA protocol provided by RayBiotech (Georgia, United States).

### 2.8 Oral glucose tolerance test (OGTT) and insulin tolerance test (ITT)

Mice were fasted for 12 h, after which a blood sample was collected from the tail tip using a glucometer (Guide, Roche, Basel, Switzerland) to measure fasting blood glucose levels. Subsequently, each mouse was administered 150 μL of a 20% glucose solution via oral gavage. Blood glucose levels were measured at 1 h and 2 h post-gavage using the aforementioned method.

After a 4–6 h fast, baseline blood glucose was measured (tail vein; time 0), followed by i. p. injection of human regular insulin (0.75–1.0 U/kg). Glucose levels were monitored at 15, 30, 45, and 60 min post-injection. Tests were terminated if glucose fell below 2.8 mmol/L, with 10% glucose administered to prevent hypoglycemia.

### 2.9 Cell culture and reagents

The rat INS-1 insulinoma cell line was obtained from the American Type Culture Collection (ATCC, Virginia, United States). Cells were cultured in 100 mm culture dishes using RPMI 1640 medium (C11875500BT, Thermo Fisher Scientific, Massachusetts, United States), supplemented with 10% fetal bovine serum and 1% penicillin-streptomycin, in a humidified incubator at 37°C with 5% CO_2_. After reaching confluence, the culture medium was aspirated, and the cells were washed three times with PBS. Subsequently, 1 mL of trypsin was added, and the cells were incubated at 37°C for 7 min to facilitate digestion. The reaction was terminated by adding 2 mL of complete culture medium, and the cells were gently pipetted to disperse. The cell suspension was collected in a 10 mL centrifuge tube and centrifuged at 1,000 rpm. The supernatant was discarded, and the cells were resuspended for subsequent passaging or plating for experimental procedures.

### 2.10 Establishment of an *in vitro* model for β-cell dedifferentiation

The culture medium in the plates was aspirated and washed with PBS. Subsequently, RPMI 1640 medium containing glucose concentrations of 20 mM, 30 mM, 40 mM, 50 mM, and 60 mM was used to induce β-cell dedifferentiation for 24, 48, and 72 h, with media changes performed daily. The optimal glucose concentration and incubation time for induction were determined using PCR analysis.

### 2.11 Ribonucleic acid (RNA) extraction and polymerase chain reaction

Cells were seeded at a density of 3 × 10^4^ cells per well in a 6-well plate and induced with high-glucose medium. Following this, the culture medium was aspirated from each well, and RNA was extracted using the cell RNA extraction kit (EZB, Wuhan, China) according to the following protocol: (1) Add 500 μL of lysis buffer to each well and incubate on a shaker at room temperature for 5 min (2) Add 500 μL of absolute ethanol, mix gently with a pipette until no precipitate remains, and transfer to a centrifuge column. Centrifuge at 12,000 g for 1 min, then discard the liquid in the collection tube. (3) Add 10 μL of ddH2O and 2 μL of gDNA remover to each sample and incubate at room temperature for 5 min (4) Add 500 μL of wash buffer, centrifuge at 12,000 g for 1 min, discard the liquid in the collection tube, and centrifuge again for 1 min. Place the centrifuge column in a 1.5 mL non-enzymatic Eppendorf tube. (5) Add 30 μL of elution buffer to the center of the centrifuge column and incubate at room temperature for 2 min. Centrifuge at 12,000 g for 1 min; the resulting liquid is RNA, which should be placed on ice.

RNA concentration was determined using a micro-volume UV spectrophotometer at OD230/260 nm. The volume of RNA required was calculated based on a target concentration of 1,000 ng/μL. Subsequently, 5 μL of 4X RT Master Mix was added to each sample, and the total reaction volume was adjusted to 20 μL with ddH2O. The mixture was thoroughly mixed and then placed in a thermal cycler, set to react at 42°C for 15 min, followed by 95°C for 30 s. The resultant solution was cDNA, which was cooled on ice and then stored at −80°C.

Real-time quantitative polymerase chain reaction (PCR) was conducted with the following cycling conditions: initial activation of the hot-start enzyme at 95°C for 10 min, followed by fluorescence signal collection. The PCR reaction consisted of denaturation at 95°C for 10 s, annealing at 60°C for 30 s, with fluorescence signal collection, repeated for 40 cycles. The temperature transition rate for both heating and cooling was set to 1.6 C/s. Primers were designed and synthesized by Shanghai Sangon Biological Engineering Co., Ltd., with the specific primer sequences listed in Table 1.

**TABLE 1:** Primer sequences.

Gene	Forward Primer (5′-3′)	Reverse Primer (5′-3′)
INS	CTG​GTG​CAG​CAC​TGA​TCT​ACA	AGC​GTG​GCT​TCT​TCT​ACA​CAC
PDX1	TGA​ACT​TGA​CCG​AGA​GAC​ACA​T	GGT​CCC​GCT​ACT​ACG​TTT​CTT​A
FOXO1	GGG​TCC​CAC​AGC​AAT​GAT​GA	TCA​AGC​GGT​TCA​TGG​CAG​AT
NGN3	GCA​GAG​CAG​ATA​AAG​CGT​GC	TCG​CCT​GGA​GTA​AAT​TGC​GT
β-Actin	GAT​TAC​TGC​CCT​GGC​TCC​TAG	GAA​AGG​GTG​TAA​AAC​GCA​GCT​C

Experimental data were calculated using the 2^−ΔΔCt^, method, where ΔΔCt = (Ct sample - Ct reference) _experimental group_ - (Ct sample - Ct reference) _control group_, with β-actin selected as the reference gene.

### 2.12 Cell transcriptomics sequencing

Following the methods outlined in Section 2.11, cells were processed and RNA was collected into centrifuge tubes. The collected RNA was then sent to Beijing Novogene Technology Co., Ltd. for transcriptomic analysis. We performed statistical analysis and data visualization using R software (version 4.3.1, R Foundation for Statistical Computing).

### 2.13 Detection of ROS levels

Following the procedures outlined in Section 2.11 for plating and cell modeling, the cells were collected into a 1.5 mL centrifuge tube and centrifuged at 1,000 rpm for 5 min. The ROS staining solution was prepared using a ROS assay kit (S0033S, Beyotime, Shanghai, China) according to the manufacturer’s instructions. The cells were then incubated at 37°C for 20 min. After incubation, the levels of ROS in the cells were observed using a flow cytometer (Agilent NovoCyte, California, United States).

Furthermore, cells were cultured using the protocol outlined in Section 2.11 and treated with the ROS-sensitive fluorescent probe DCFH-DA (S0033S, Beyotime, Shanghai, China) as per the manufacturer’s guidelines. Fluorescence imaging was then performed using an inverted fluorescence microscope.

### 2.14 Detection of superoxide dismutase (SOD), malondialdehyde (MDA) and glutathione (GSH) levels

Following the procedures outlined in Section 2.11 for plating and establishing the *in vitro* dedifferentiation model, SOD, MDA and GSH levels were measured according to the manufacturer’s instructions (S0101S, S0131M, S0057S, Beyotime, Shanghai, China). Subsequently, the protein concentrations of each group of samples were determined using a BCA assay kit (23,225, Thermo Fisher, Massachusetts, United States), and the concentrations of SOD and MDA in each group were calculated accordingly. A concentration of 10 μM glimepiride (GM) was employed as the positive control in these studies.

### 2.15 Observation of the endoplasmic reticulum

Cells were seeded at a density of 2000 cells per well in confocal dishes (150,680, Thermo Fisher, Massachusetts, United States) and the dedifferentiation model was established following the method described in Section 2.10. The endoplasmic reticulum was labeled using ER-Tracker Green (C1041S, Beyotime, Shanghai, China), and its morphology was observed using a laser confocal microscope (LSM900, Zeiss, Oberkochen, Germany).

### 2.16 Observation of cell morphology

Following the procedures outlined in Section 2.10 for plating and establishing the *in vitro* dedifferentiation model, cell morphology was observed using an inverted microscope.

### 2.17 Immunofluorescence

Following the procedures outlined in Section 2.11 for plating and establishing the *in vitro* dedifferentiation model, the culture medium was aspirated and the cells were washed three times with PBS. Cells were fixed using 4% formaldehyde solution and permeabilized with 0.5% Triton solution. The cells were incubated with BSA solution for 1 h, followed by overnight incubation with the primary antibody at 4°C. After recovering the primary antibody, the cells were washed three times with PBS and incubated with the fluorescent secondary antibody for 1 h. Fluorescence intensity was observed using an inverted fluorescence microscope (Canon, Japan).

### 2.18 Western blot

Following the procedures outlined in Section 2.11 for plating and establishing the *in vitro* dedifferentiation model, the culture medium was aspirated and the cells were washed three times with PBS. Then, 50 μL of RIPA buffer (Biyuntian, Shanghai, China), supplemented with protease and phosphatase inhibitors, was added to each well and incubated on ice at 80 rpm for 30 min. The cell lysates were collected using a cell scraper into 1.5 mL centrifuge tubes and centrifuged at 4°C at 13,200 rpm for 15 min. The supernatant was extracted, and protein concentration was measured using a BCA assay kit (Thermo Fisher, United States). Samples were mixed with 5X loading buffer and denatured at 100°C for 10 min.

### 2.19 Data analysis

Statistical analysis of the experimental results was performed using SPSS 22.0 software. For quantitative data, the Shapiro-Wilk test was used to assess normality. If the data followed a normal distribution, results were expressed as mean ± standard deviation ( $\bar{x}$ ± s), and comparisons between groups were conducted using one-way ANOVA. If the data did not meet the normality assumption, the median M (P25-P75) was used to describe the results, and group comparisons were performed using the Kruskal–Wallis rank-sum test. If the data were normally distributed and met the assumption of homogeneity of variance, multiple comparisons were adjusted using the Bonferroni correction. If the data were normally distributed but did not meet the homogeneity of variance assumption, Dunnett’s T3 test was used. A significance level of *P* < 0.05 was considered statistically significant.

## 3 Results

### 3.1 Effects of Op D on β-cell dedifferentiation in mouse models

The research team established a dedifferentiation mouse model and intervened with Op D. The results showed that, compared to the Mm group, Op D significantly reduced body weight and blood glucose levels, with the effect being most pronounced in the high-dose group (Figures 2B,C). Both the insulin tolerance test (ITT) and Oral glucose tolerance test (OGTT) experiments confirmed that Op D improved glucose tolerance in the mice (Figures 2D,E). Subsequent measurements of serum insulin and glucagon levels indicated that Op D promoted insulin release but did not reduce glucagon levels (Figures 2F,G). Given that diabetes is often associated with lipid metabolism abnormalities, we also monitored serum triglyceride (TG) and cholesterol (CHOL) levels in the mice. The results demonstrated that Op D treatment significantly decreased serum TG and CHOL levels in diabetic mice (Figures 2H,I). HE staining revealed that diabetic mice exhibited marked pancreatic atrophy; however, treatment with Op D significantly ameliorated this atrophy (Figure 2J).

### 3.2 Effects of Op D on the expression levels of dedifferentiation-related proteins in mouse pancreas

**FIGURE 3:**
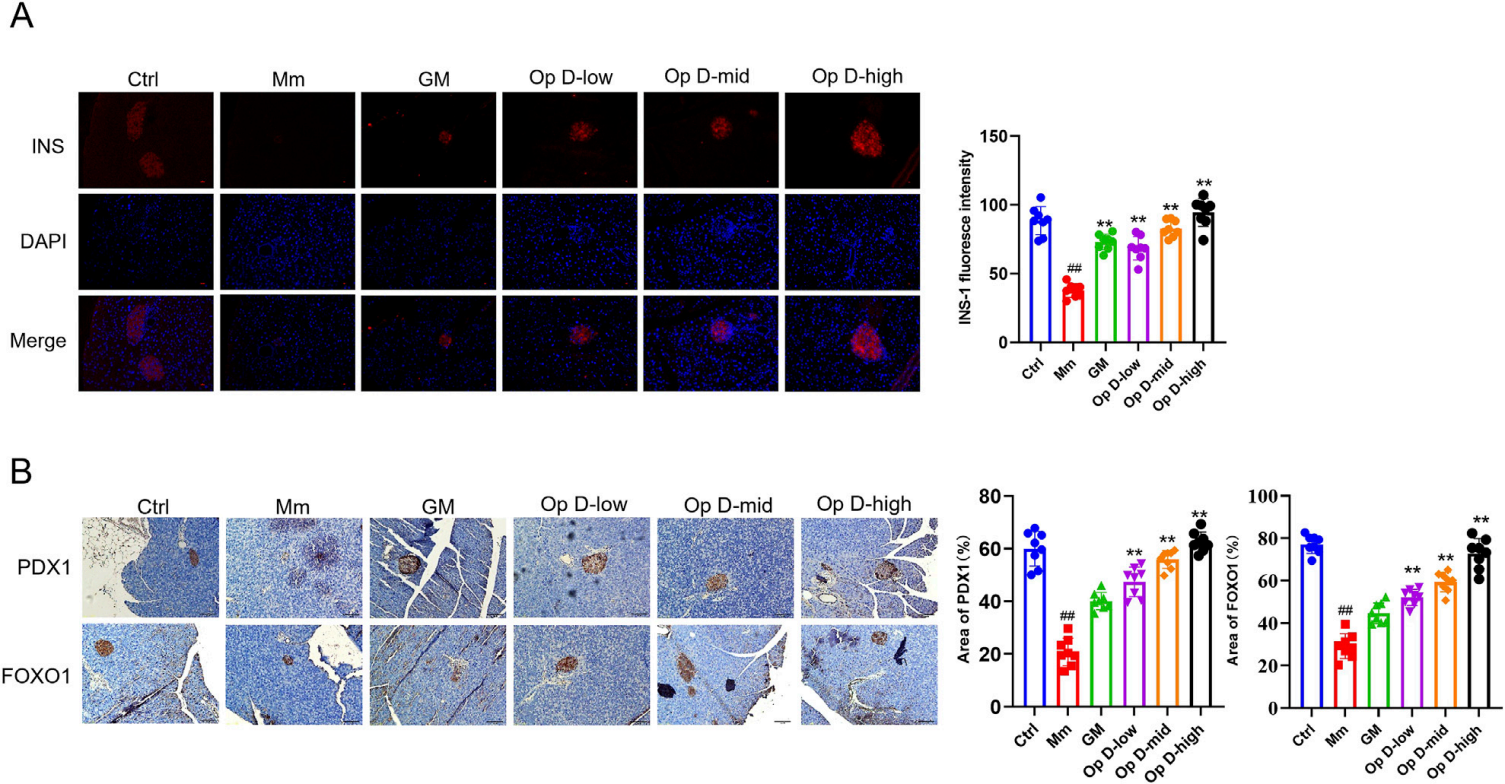
Effects of Op D on the expression levels of dedifferentiation-relatedproteins in diabetic mice. **(A)** Immunofluorescence results of INS-1 protein in mouse pancreatic tissue. Scale bar = 20 μM; **(B)** Immunohistochemistry and statistical results of PDX1 and FOXO1 in mouse pancreatic tissue (n = 7). Scale bar = 50 μM, 20 μM; *P < 0.05, **P < 0.01 versus Mm; #*P* < 0.05, ##*P* < 0.01 versus Ctrl. In Figure B, the vertical axis of the statistical graph represents the “area,” which refers to the percentage of the stained region area relative to the total field of view area.

We first used immunofluorescence to detect the levels of INS in pancreatic tissue. The results showed that the INS levels in the Mm group were significantly reduced. However, after intervention with Op D, the INS levels in the islets of mice were significantly increased (Figure 3A), with the high-dose group showing the most pronounced effect. Subsequently, we further examined the expression levels of PDX1 and FOXO1 in the mouse pancreas. The results indicated that the expression levels of PDX1 and FOXO1 in the Mm group were significantly decreased, while Op D intervention increased the expression levels of PDX1 and FOXO1 (Figure 3B), with the high-dose group being the most significant.

### 3.3 Effects of different glucose concentrations on INS-1 cells and the expression levels of dedifferentiation-related genes at different time points

**FIGURE 4:**
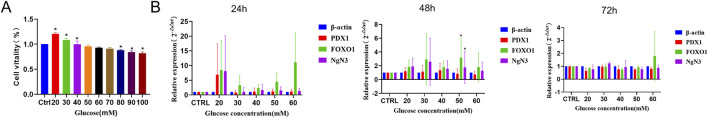
Effects of different glucose concentrations on INS-1 cell proliferation and the expression levels of PDX1, FOXO1, and NgN3 mRNA at different time points. **(A)** Effects of different glucose concentrations on INS-1 cell proliferation. **(B)** Effects of different glucose concentrations on the expression levels of PDX1, FOXO1, and NgN3 mRNA at different time points (n = 6). *P < 0.05 versus Ctrl.

To establish a cell model of INS-1 cell dedifferentiation, we incubated INS-1 cells with different concentrations of glucose for 24 h. As shown in Figure 4A, glucose concentrations of 20 mM and 30 mM promoted cell proliferation, while glucose concentrations of 80 mM, 90 mM, and 100 mM significantly inhibited INS-1 cell proliferation. Therefore, we selected glucose concentrations below 60 mM to explore their effects on the expression levels of dedifferentiation-related genes in the cells. Our subsequent investigations further elucidated the temporal effects of high glucose exposure on β-cell dedifferentiation (Figure 4B). The results demonstrated that prolonged exposure to 50 mM glucose for 48 h significantly elevated NgN3 expression levels, ultimately triggering β-cell dedifferentiation. Based on these findings, we established an *in vitro* experimental model of β-cell dedifferentiation using 48-h high glucose induction.

### 3.4 Cytotoxicity of Op D and its effects on INS-1 cell dedifferentiation

**FIGURE 5:**
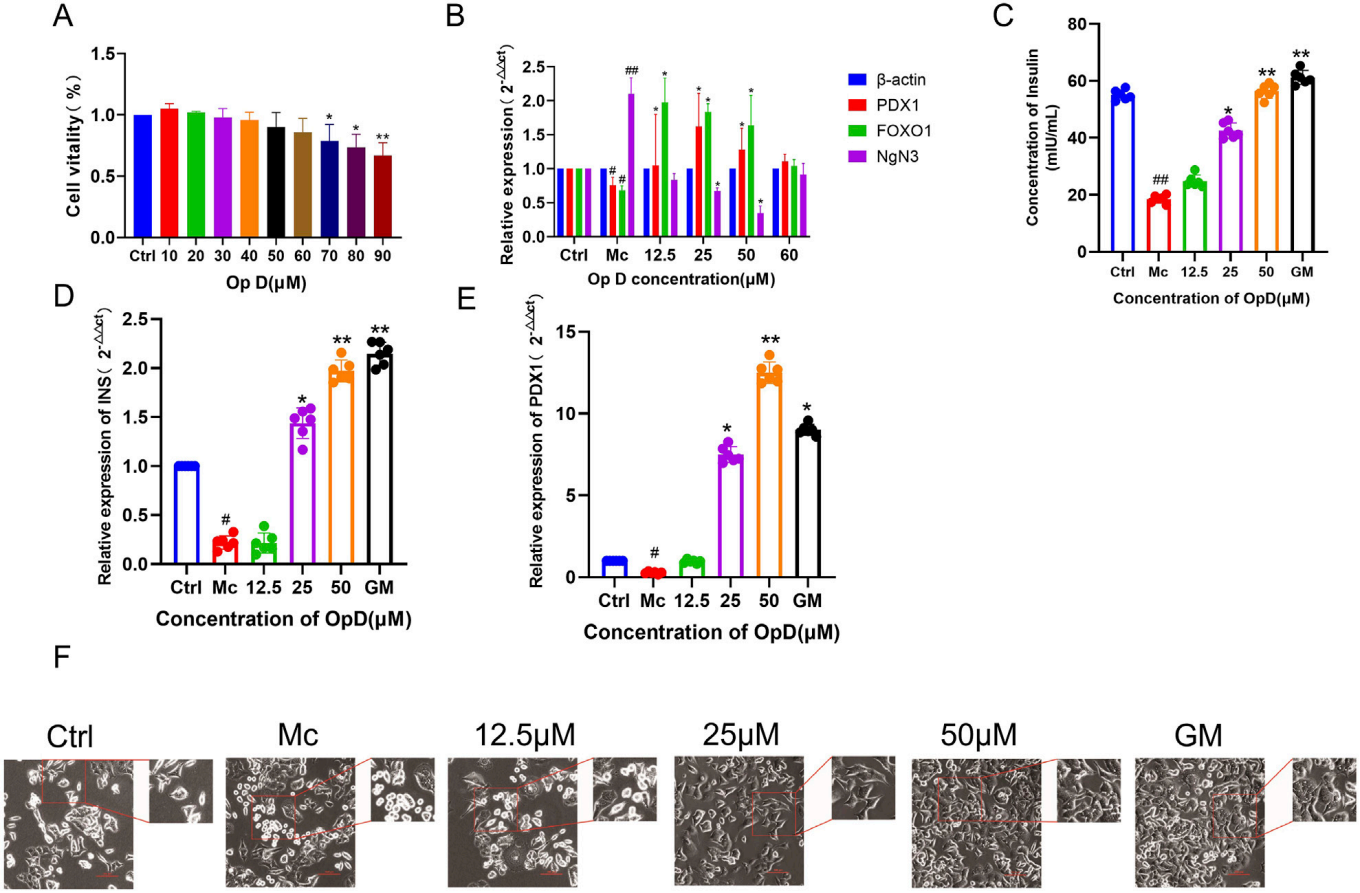
Effects of Op D on the proliferative activity, morphology, and function of INS-1 cells. **(A)** Effects of different concentrations of Op D on cell proliferative activity; **(B)** Effects of different concentrations of Op D on the mRNA expression levels of dedifferentiation-related genes; **(C)** Insulin secretion levels in INS-1 cells; **(D)** mRNA expression levels of INS in cells; **(E)** Expression levels of PDX1 in cells; **(F)** Effects of Op D on the morphology of INS-1 cells (n = 6). Bar, 50 μm *P < 0.05, **P < 0.01 versus Mc; ##*P* < 0.01 versus Ctrl.

To further investigate the effects of Op D on INS-1 cell dedifferentiation, we initially determined its safety range using the MTT assay. As presented in Figure 5A, Op D concentrations below 70 μM showed no significant impact on INS-1 cell proliferation. We subsequently examined the influence of 12.5 μM, 25 μM, 50 μM, and 60 μM Op D on the expression of dedifferentiation-associated regulatory genes in INS-1 cells. The experimental data demonstrated that 50 μM Op D markedly elevated PDX1 and FOXO1 levels while reducing NgN3 expression (Figure 5B). Notably, 60 μM Op D did not produce significant alterations in the mRNA expression levels of PDX1, FOXO1, or NgN3. Consequently, we employed Op D concentrations of 12.5 μM, 25 μM, and 50 μM for subsequent experimental procedures. Further investigations revealed that 50 μM Op D significantly enhanced both insulin secretion and INS gene mRNA expression in INS-1 cells (Figures 5C,D). Additionally, Op D treatment upregulated PDX-1 gene mRNA levels in these cells (Figure 5E). Morphological analysis (Figure 5F) indicated that the model group exhibited a predominance of rounded cellular morphology, whereas Op D intervention promoted the restoration of spindle-shaped cellular morphology, substantially decreasing the proportion of rounded cells in the microscopic field.

### 3.5 Transcriptomic exploration of potential pathways by which op D improves INS-1 cell dedifferentiation

**FIGURE 6:**
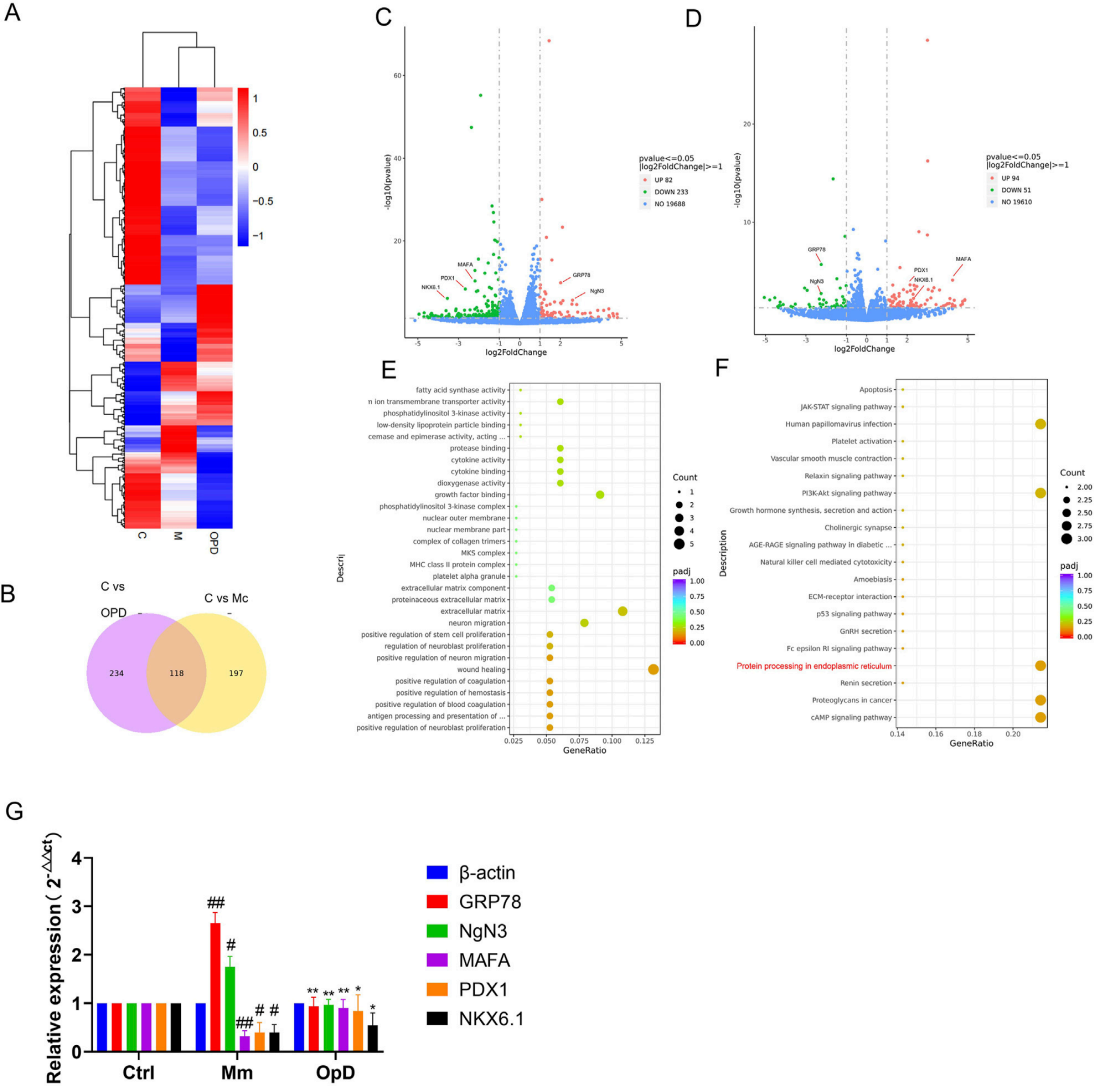
Mechanisms by which Op D improves INS-1 dedifferentiation based on transcriptomics. **(A)** Heatmap of mRNA expression differences among Ctrl, M, and Op D groups; **(B)** Venn diagram; **(C)** Heatmap of differentially expressed genes between the Ctrl group and the M group; **(D)** Heatmap of differentially expressed genes between the M group and the Op D group; **(E)** GO enrichment plot of differentially expressed genes in the Op D group; **(F)** KEGG enrichment plot of differentially expressed genes in the Op D group; **(G)** Analysis of mRNA expression levels for GRP78, NKX6.1, PDX1, MAFA, and NGN3 (n = 6). *P < 0.05, **P < 0.01 versus Mc; ##*P* < 0.01 versus Ctrl.

To further explore the potential mechanisms by which Op D improves pancreatic β-cell dedifferentiation, we performed transcriptomic analysis of differentially expressed genes. The results showed that there were 315 differentially expressed genes between the M group and the Ctrl group, with 82 genes upregulated and 233 (|log2FC|>1, *P* ≤ 0.05) genes downregulated. Comparing the model group with the Op D group, there were 145 differentially expressed genes, with 51 genes upregulated and 93 genes downregulated (|log2FC|>1, *P* ≤ 0.05), among which GRP78 (|log2FC| = 1.912, -log10 (*p*value) = 16.284), NgN3 (|log2FC| = 2.845, -log10 (*p*value) = 7.416), NKX6.1 (|log2FC| = 3.529, -log10 (*p*value) = 8.223), PDX1 (|log2FC| = 2.745, -log10 (*p*value) = 10.140) and MAFA (|log2FC| = 2.377, -log10 (*p*value) = 16.408) showed significant differences (Figures 6A-D). Subsequently, we conducted KEGG enrichment analysis of the differentially expressed genes, revealing that the protein processing in endoplasmic reticulum pathway was the most significantly enriched. MAF bZIP transcription factor (Figures 6E,F). MAFA (MAF bZIP transcription factor A) is highly expressed in terminally differentiated cells such as pancreatic β-cells, where it plays a critical role in maintaining their mature functions and regulating the expression of insulin gene. The loss of MAFA leads to reduced insulin secretion and decreased expression of β-cell markers (e.g., PDX1), resulting in the reversion of cells to an immature, less differentiated state. Loss of NKX6.1 leads to decreased expression of key β-cell genes (such as INS, MAFA, and PDX1), resulting in the loss of mature cellular function. These genes are critical markers of β-cell identity, and their downregulation serves as a key indicator of β-cell dedifferentiation (Zhu et al., 2017). Therefore, OpD treatment significantly altered the mRNA expression levels of NgN3, MAFA, PDX1, and NKX6.1, effectively ameliorating the dedifferentiation phenotype in INS1 cells (Figure 6G).

### 3.6 Effects of Op D on ROS, SOD, MDA, and the endoplasmic reticulum stress in INS-1 cells

**FIGURE 7:**
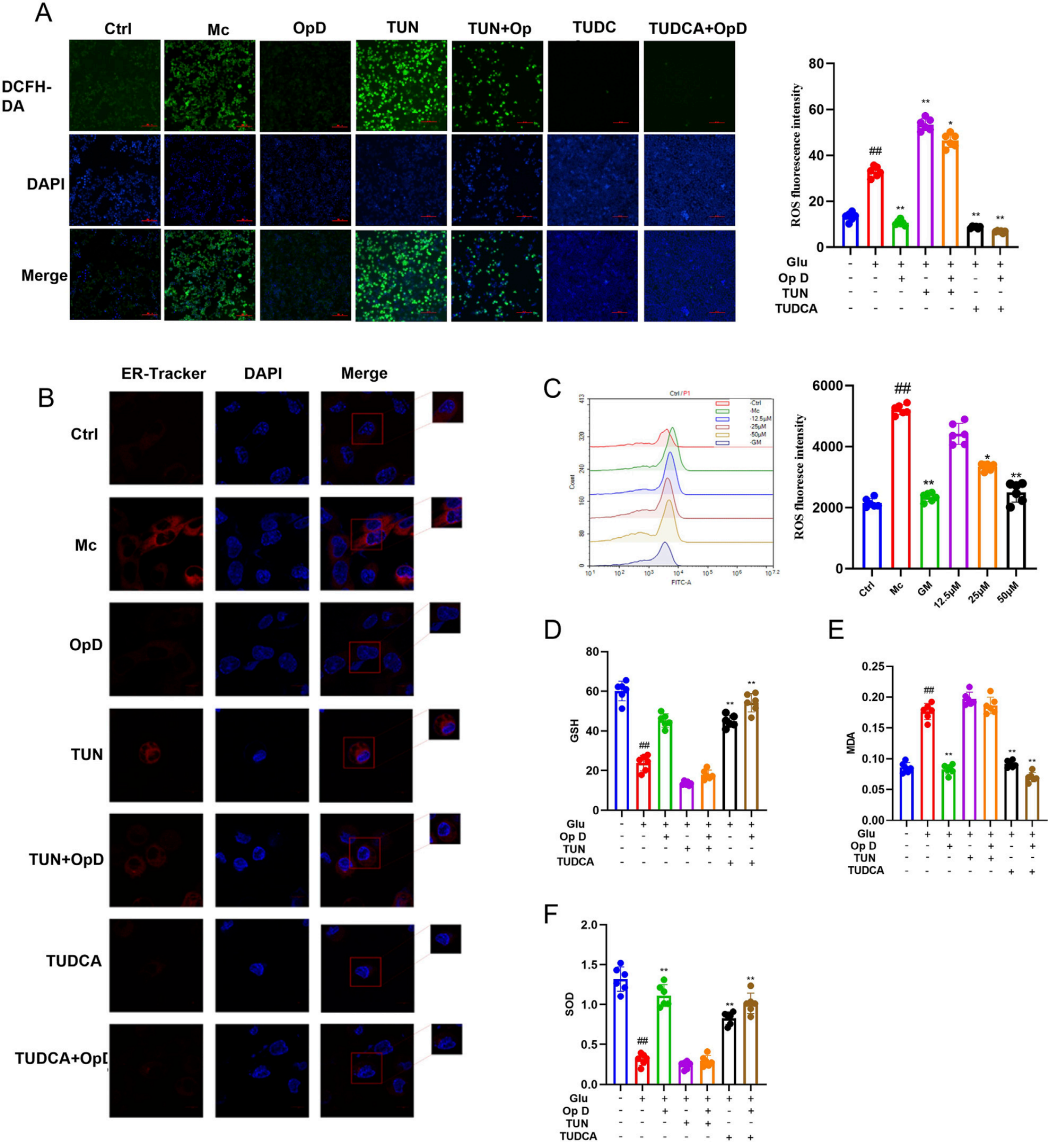
Effects of Op D on ROS, endoplasmic reticulum, GSH, MDA, and SOD in INS-1 cell lines. **(A)** Fluorescence images and statistical results of intracellular ROS levels (Scale bar 50 μM); **(B)** Confocal microscopy observation of the endoplasmic reticulum in cells (Scale bar 10 μM); **(C)** Flow cytometry images and statistical results of ROS; **(D)** Intracellular GSH levels; **(E)** Intracellular MDA levels; **(F)** Intracellular SOD levels (n = 6). *P < 0.05, **P < 0.01 versus Mc; ##*P* < 0.01 versus Ctrl. TUN refers to the simultaneous treatment with high glucose and Tunicamycin; TUN + Op D means the combined use of Tunicamycin and Op D after high glucose treatment; TUDCA refers to the simultaneous treatment with high glucose and tauroursodeoxycholic acid; TUDCA + Op D means the combined use of tauroursodeoxycholic acid and Op D after high glucose treatment.

Based on transcriptomic results, the protein processing pathway in the endoplasmic reticulum (ER) may be a crucial mechanism by which Op D improves pancreatic β-cell dedifferentiation. The unfolded protein response in the ER is closely related to ER stress, which can produce a large amount of oxidative stress products. Therefore, we measured the levels of ROS in the cells. As shown in Figures 7A,C, the ROS levels in the model group were significantly increased, while the application of Op D effectively reduced the intracellular ROS levels, with 50 μM Op D showing the most pronounced effect. Consequently, in subsequent experiments, we used this concentration of Op D in combination with an ER stress inhibitor (Tauroursodeoxycholic acid, TUNCA) and an agonist (Tunicamycin, TUN) to further clarify the potential mechanisms of Op D. The doses of TUN and TUNCA are detailed in Supplementary File S2.

We further used ER-Tracker to label the ER in cells and observed them under a laser confocal microscope. The results showed that the ER in the model group exhibited large vacuolar structures, while the application of Op D reduced the ER vacuoles. However, the effect of Op D was weakened when the ER stress agonist was applied (Figure 7B). We then measured the levels of MDA, GSH, and SOD. The results showed that MDA levels were significantly increased in the model group, while GSH and SOD levels were decreased. The application of Op D reversed these changes, with more significant effects when combined with the ER stress inhibitor, but the effects were weakened when combined with the agonist (Figures 7D-F). These findings suggest that the improvement of pancreatic β-cell dedifferentiation by Op D may be related to the inhibition of ER stress and oxidative stress levels in cells.

### 3.7 Effects of Op D on the expression levels of ER stress-related proteins and pancreatic β-cell dedifferentiation-related proteins in cells

**FIGURE 8:**
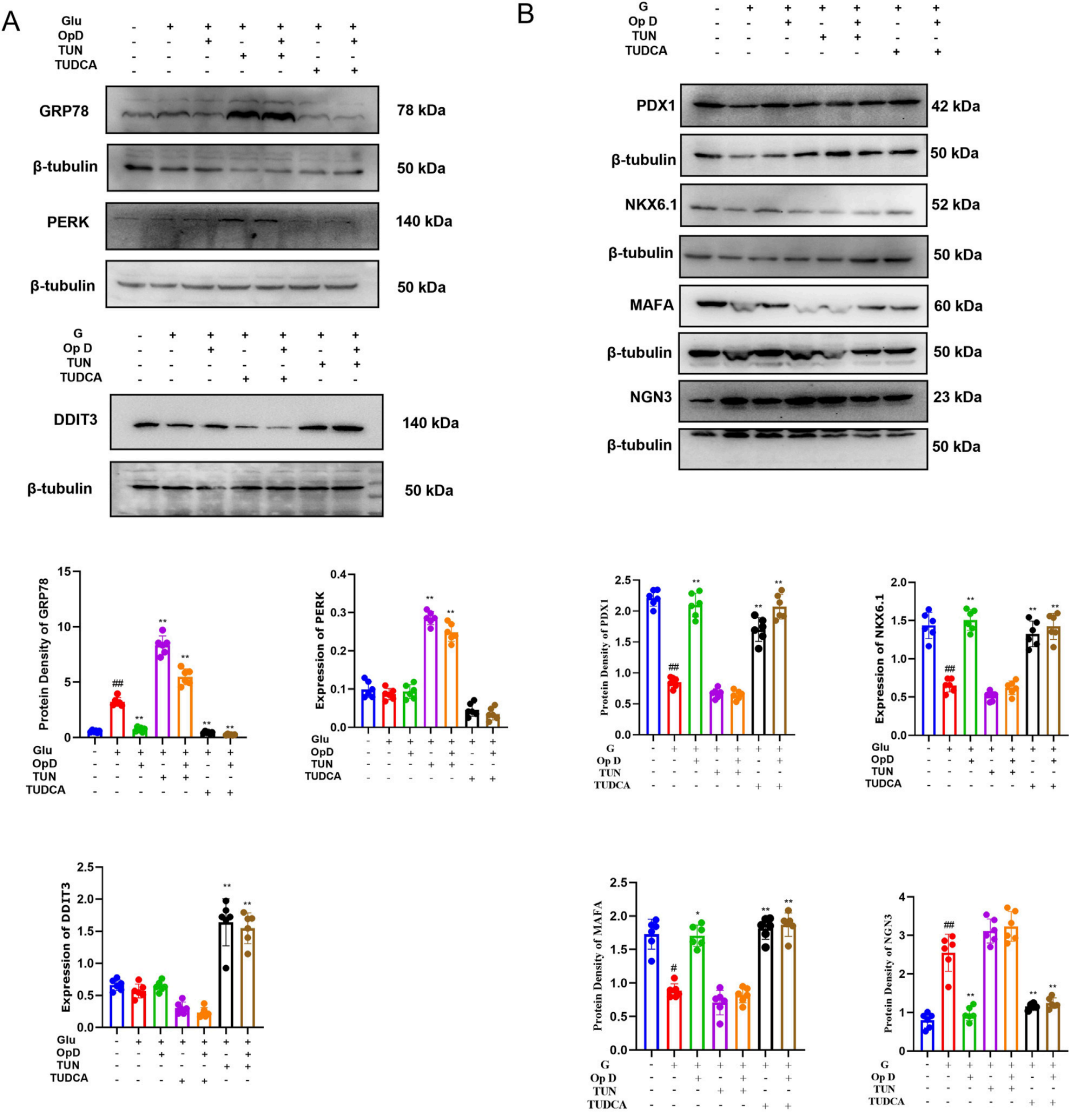
Mechanisms by which Op D improves pancreatic β-Cell dedifferentiation. **(A)** Western blot bands and statistical analysis of ER stress-related proteins GRP78, PERK, and DDIT3; **(B)** Western blot bands and statistical analysis of dedifferentiation-related proteins PDX1, MAFA, NGN3, and NKX6.1; **(C)** Immunofluorescence results and statistical analysis of FOXO1 and PDX1 (n = 6). *P < 0.05, **P < 0.01 versus Mc; ##*P* < 0.01 versus Ctrl.

To further elucidate the potential mechanism by which Op D improves pancreatic β-cell dedifferentiation by inhibiting ER stress, we used Western blot (WB) to monitor the protein expression levels of relevant factors. The results showed that the application of Op D reduced the levels of GRP78 in cells. However, when an ER stress agonist was applied, Op D no longer had an effect. Additionally, Op D did not affect the expression of DDIT3 and PERK (Figure 8A).

Subsequently, we further examined the expression levels of ER stress-related proteins in cells. Administration of Ophiopogonin D (Op D) downregulated the expression levels of GRP78 and PERK, whereas this suppressive effect was abolished upon co-treatment with an ER stress agonist. As demonstrated by WB analysis and quantitative data in Figure 8B, Op D upregulated PDX1, NKX6.1, and MAFA while suppressing NgN3 expression, collectively ameliorating β-cell dedifferentiation. However, the therapeutic efficacy of Op D in reversing β-cell dedifferentiation was negated when ER stress was pharmacologically exacerbated by the agonist.

Furthermore, PDX1 can translocate from the cytoplasm to the nucleus to regulate transcription. Therefore, we used immunofluorescence to observe the distribution of PDX1 under a confocal microscope. The results indicated that in dedifferentiated cells, the fluorescence of PDX1 in the nucleus decreased. However, after Op D treatment, the distribution of PDX1 in the nucleus increased, while the ER stress agonist limited the effect of Op D. Additionally, immunofluorescence showed that the application of Op D increased the expression level of FOXO1 in cells.

## 4 Discussion

The number of people affected by diabetes is increasing annually, making it one of the diseases that seriously endangers human health. Diabetes is characterized by high disability and mortality rates, which can impose a significant economic burden on patients’ families and society. Current research suggests that mature pancreatic β-cells can dedifferentiate into endocrine progenitor cells, leading to the loss of β-cell function, reduced insulin secretion, and the progression of diabetes (Hogrebe et al., 2021). However, this process can be reversed, making it a new target for diabetes treatment. Previous studies have shown that Op D can improve symptoms in diabetic animal models (Qiao et al., 2020), but its mechanism of action on β-cell dedifferentiation has not been confirmed. Therefore, this study aims to further explore the effects of Op D on β-cell dedifferentiation.

Ophiopogon japonicus (Thunb.) Ker Gawl., a traditional Chinese medicinal herb, has potential value as a health food. Op D, a steroidal saponin extracted from the rhizomes of Ophiopogon japonicus, possesses various biological activities, including anti-inflammatory, anti-cancer, renal protective, and cardioprotective effects (Zang et al., 2016; Qiao et al., 2020; Ko et al., 2022; Wang et al., 2022). Previous studies have shown that Op D can reduce blood glucose and lipid levels in a diabetic cardiomyopathy mouse model (Li et al., 2021). In our study, we found that the application of Op D reduced blood glucose and lipid levels in diabetic mice, improved ITT and OGTT in mice, and significantly slowed the weight gain of mice in the high-dose group compared to the model group during the experimental period. Pathological staining showed that Op D significantly improved the atrophy of islets in pancreatic tissue. Further IHC and immunofluorescence experiments indicated that the application of Op D increased the expression levels of INS, PDX1, and FOXO1 in pancreatic tissue, demonstrating the potential of Op D to regulate β-cell dedifferentiation.

The differentiation and maturation of β-cells are regulated by the timely expression levels of a complex network of transcription factors (TFs) such as PDX1, NEUROG3, NKX6.1, and FOXO1 (Salinno et al., 2019). In experimentally induced hyperglycemic rodent models, islets lose the expression of key β-cell-specific TFs and maturation markers (Laybutt et al., 2002; Laybutt et al., 2003). Researchers have found that in FOXO1-deficient mouse pancreases, β-cells do not undergo apoptosis, but their β-cell maturation markers and TFs are downregulated, while TFs marking pluripotency or endocrine progenitor cells (Neurog3, Oct4, Nanog) are upregulated (Gao et al., 2015).

INS-1 cells, derived from rat insulinoma cells, can be used to establish *in vitro* models of diabetes. In this experiment, we used 50 mM glucose to induce INS-1 cells to establish a model of islet cell dedifferentiation. Studies have shown that Op D can improve the apoptosis of INS-1 cells induced by hydrogen peroxide, thereby improving their function (Ko et al., 2022). Our study found that after 48 h of high glucose induction, INS-1 cells did not undergo apoptosis. Microscopically, the morphology of cells in the model group shifted towards progenitor cells, while Op D improved this phenomenon by increasing the mRNA levels of FOXO1 and PDX1 and decreasing the mRNA level of NgN3.

Subsequently, we used transcriptomic analysis to further clarify the potential mechanisms by which Op D improves β-cell dedifferentiation. The results indicated that the protein processing pathway in the endoplasmic reticulum (ER) might be the primary mechanism of Op D’s action, with GPR68 being a potential key target. The ER is crucial for the synthesis and folding of secretory and membrane proteins (Depaoli et al., 2019). Approximately one-third of cellular proteins are transferred into the ER lumen, where molecular chaperones and enzymes mediate their folding into three-dimensional structures (Wang et al., 2025). Insulin, as a secretory protein, cannot be properly folded and matured within the endoplasmic reticulum (ER). When the demand for protein folding exceeds the ER’s folding capacity, the overload triggers the collapse of the protein quality control system, ultimately resulting in endoplasmic reticulum stress. (Ghosh et al., 2019). The accumulation of misfolded proteins can trigger ER stress, and this adaptive protein folding response releases GRP78 to prevent the aggregation of these misfolded proteins and promote their correct folding. The release of GRP78 is also considered an important marker of ER stress (Eizirik et al., 2023; Liu et al., 2023). ER stress-mediated oxidative stress is closely related to subsequent β-cell dedifferentiation (Luo et al., 2022). The identity specification of insulin-producing β-cells is initiated during the primary transition phase of the pancreatic endodermal bud, a process coordinately regulated through combinatorial interactions among transcription factors such as pancreatic and duodenal homeobox 1 (PDX1) and the NK6 homeodomain factor NKX6.1 (Weidemann et al., 2024). PDX1 further promotes β-cell ontogeny by suppressing the α-cell gene network (including networks governed by the Aristaless-related homeobox, ARX) (Dhawan et al., 2011; Gao et al., 2014) and repressing factors that impair aerobic metabolism, such as carboxylate transporters. Our experimental results demonstrated that Ophiopogonin D suppresses GPR68 and PERK to inhibit high glucose-induced endoplasmic reticulum stress (ERS), while upregulating the expression of proteins such as DDIT3, PDX1, and NKX6.1, thereby reversing the dedifferentiation of pancreatic β-cells. However, upon administration of an ER stress agonist, the inhibitory effects of Ophiopogonin D on GPR68 and PERK were attenuated, and its ability to ameliorate β-cell dedifferentiation was abolished. Therefore, Op D may serve as a potential targeted agent to ameliorate endoplasmic reticulum stress, thereby reversing β-cell dedifferentiation, enhancing insulin secretion, and ultimately improving diabetic pathology.

## 5 Conclusion

**FIGURE 9:**
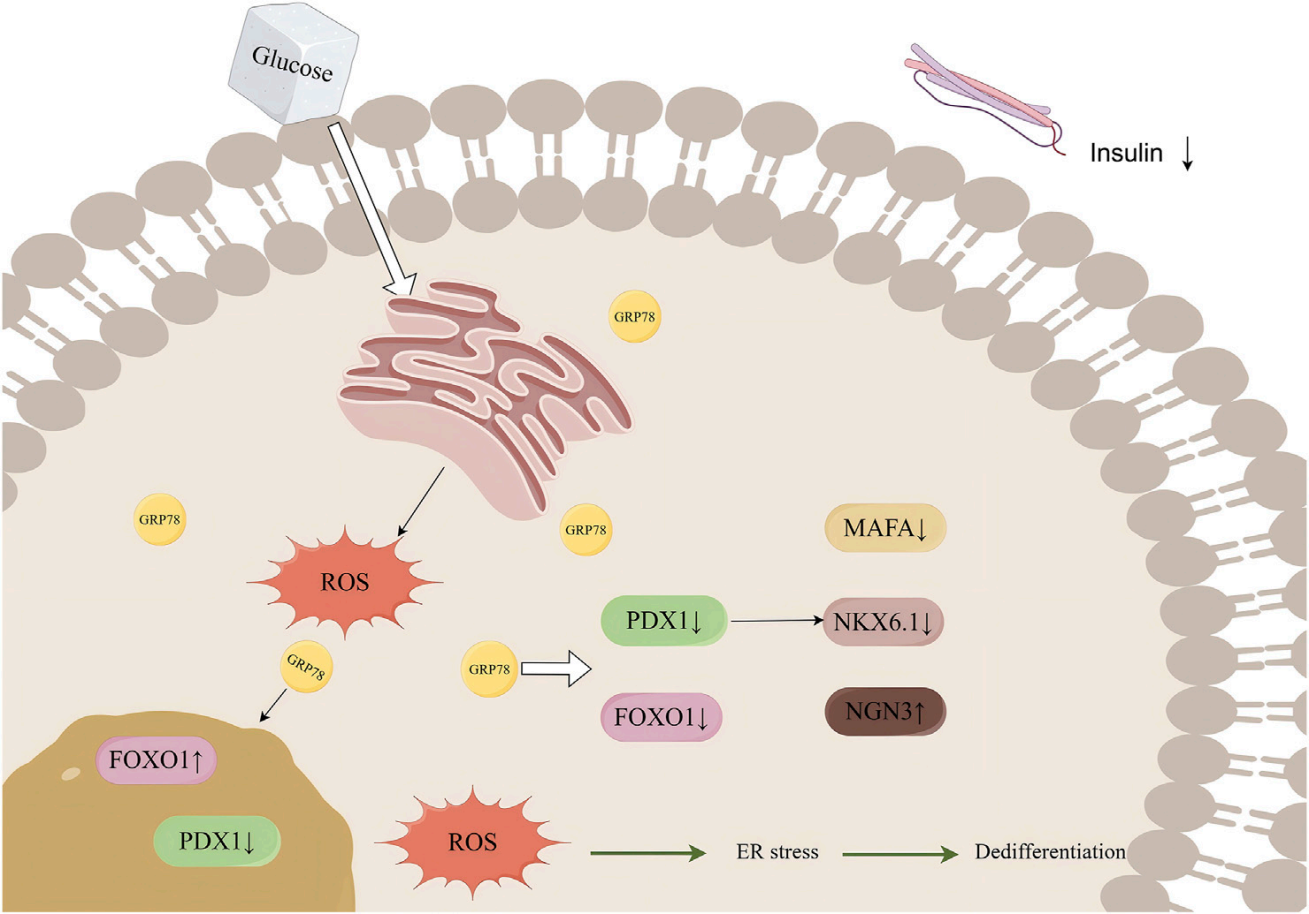
Mechanism diagram.

Op D could improve pancreatic β-cell dedifferentiation by reducing intracellular oxidative stress through the inhibition of endoplasmic reticulum stress, thereby lowering blood glucose levels and improving glucose tolerance in mice. This effect may be mediated through the regulation of the GRP78/ROS/FOXO1 axis (Figure 9).

## Data Availability

The original contributions presented in the study are included in the article/Supplementary Material, further inquiries can be directed to the corresponding authors.

## Abbreviations

American Type Culture Collection, ATCC, cholesterol; CHOL; diabetes mellitus, DM; endoplasmic reticulum, ER; Forkhead box O1, FOXO1; glimepiride group, GM; Hematoxylin-eosi, HE; malondialdehyde, MDA; Neurogenin3, NGN3; Oral glucose tolerance test, OGTT; Ophiopogonin D, Op D; pancreatic/duodenal homeobox 1, PDX-1; polymerase chain reaction, PCR)phosphate buffer saline, PBS; reactive oxygen species, ROS; Ribonucleic acid, RNA; superoxide dismutase, SOD; specific pathogen-free, SPF; streptozotocin, STZ; Tauroursodeoxycholic acid, TUNCA; type 2 diabetes mellitus, T2DM; triglycerides, TG; Tunicamycin, TUN; transcription factors, TFs; unfolded protein response, UPR.

## References

[B1] Bensellam M. Laybutt D. R. Jonas J. C. (2012). The molecular mechanisms of pancreatic β-cell glucotoxicity: recent findings and future research directions. Mol. Cell Endocrinol. 364 (1-2), 1–27. 10.1016/j.mce.2012.08.003 22885162

[B2] Bilekova S. Sachs S. Lickert H. (2021). Pharmacological targeting of endoplasmic reticulum stress in pancreatic beta cells. Trends Pharmacol. Sci. 42 (2), 85–95. 10.1016/j.tips.2020.11.011 33353789

[B3] Chen K. Q. Wang S. Z. Lei H. B. Liu X. (2024). Ophiopogonin D: review of pharmacological activity. Front. Pharmacol. 15, 1401627. 10.3389/fphar.2024.1401627 39101149 PMC11295246

[B4] Depaoli M. R. Hay J. C. Graier W. F. Malli R. (2019). The enigmatic ATP supply of the endoplasmic reticulum. Biol. Rev. Camb Philos. Soc. 94 (2), 610–628. 10.1111/brv.12469 30338910 PMC6446729

[B5] Dhawan S. Georgia S. Tschen S. I. Fan G. Bhushan A. (2011). Pancreatic β cell identity is maintained by DNA methylation-mediated repression of Arx. Dev. Cell 20 (4), 419–429. 10.1016/j.devcel.2011.03.012 21497756 PMC3086024

[B6] Eizirik D. L. Szymczak F. Mallone R. (2023). Why does the immune system destroy pancreatic β-cells but not α-cells in type 1 diabetes? Nat. Rev. Endocrinol. 19 (7), 425–434. 10.1038/s41574-023-00826-3 37072614

[B7] Federation I. D. (2022). 11th edition of the IDF diabetes Atlas.

[B8] Gao M. Estel K. Seeliger J. Friedrich R. P. Dogan S. Wanker E. E. (2015). Modulation of human IAPP fibrillation: cosolutes, crowders and chaperones. Phys. Chem. Chem. Phys. 17 (13), 8338–8348. 10.1039/c4cp04682j 25406896

[B9] Gao T. McKenna B. Li C. Reichert M. Nguyen J. Singh T. (2014). Pdx1 maintains β cell identity and function by repressing an α cell program. Cell Metab. 19 (2), 259–271. 10.1016/j.cmet.2013.12.002 24506867 PMC3950964

[B10] GBD 2021 Diabetes Collaborators (2023). Global, regional, and national burden of diabetes from 1990 to 2021, with projections of prevalence to 2050: a systematic analysis for the Global Burden of Disease Study 2021. Lancet 402 (10397), 203–234. 10.1016/s0140-6736(23)01301-6 37356446 PMC10364581

[B11] Ghosh R. Colon-Negron K. Papa F. R. (2019). Endoplasmic reticulum stress, degeneration of pancreatic islet β-cells, and therapeutic modulation of the unfolded protein response in diabetes. Mol. Metab. 27s (Suppl. l), S60–s68. 10.1016/j.molmet.2019.06.012 31500832 PMC6768499

[B12] Hagenlocher C. Siebert R. Taschke B. Wieske S. Hausser A. Rehm M. (2022). ER stress-induced cell death proceeds independently of the TRAIL-R2 signaling axis in pancreatic β cells. Cell Death Discov. 8 (1), 34. 10.1038/s41420-022-00830-y 35075141 PMC8786928

[B13] Hogrebe N. J. Maxwell K. G. Augsornworawat P. Millman J. R. (2021). Generation of insulin-producing pancreatic β cells from multiple human stem cell lines. Nat. Protoc. 16 (9), 4109–4143. 10.1038/s41596-021-00560-y 34349281 PMC8529911

[B14] Kamagate A. Kim D. H. Zhang T. Slusher S. Gramignoli R. Strom S. C. (2010). FoxO1 links hepatic insulin action to endoplasmic reticulum stress. Endocrinology 151 (8), 3521–3535. 10.1210/en.2009-1306 20501674 PMC2940535

[B15] Ko H. M. Jee W. Lee D. Jang H. J. Jung J. H. (2022). Ophiopogonin D increase apoptosis by activating p53 via ribosomal protein L5 and L11 and inhibiting the expression of c-Myc via CNOT2. Front. Pharmacol. 13, 974468. 10.3389/fphar.2022.974468 36569330 PMC9780504

[B16] Kopp M. C. Larburu N. Durairaj V. Adams C. J. Ali M. M. U. (2019). UPR proteins IRE1 and PERK switch BiP from chaperone to ER stress sensor. Nat. Struct. Mol. Biol. 26 (11), 1053–1062. 10.1038/s41594-019-0324-9 31695187 PMC6858872

[B17] Kupsco A. Schlenk D. (2015). Oxidative stress, unfolded protein response, and apoptosis in developmental toxicity. Int. Rev. Cell Mol. Biol. 317, 1–66. 10.1016/bs.ircmb.2015.02.002 26008783 PMC4792257

[B18] Laybutt D. R. Glandt M. Xu G. Ahn Y. B. Trivedi N. Bonner-Weir S. (2003). Critical reduction in beta-cell mass results in two distinct outcomes over time. Adaptation with impaired glucose tolerance or decompensated diabetes. J. Biol. Chem. 278 (5), 2997–3005. 10.1074/jbc.M210581200 12438314

[B19] Laybutt D. R. Sharma A. Sgroi D. C. Gaudet J. Bonner-Weir S. Weir G. C. (2002). Genetic regulation of metabolic pathways in beta-cells disrupted by hyperglycemia. J. Biol. Chem. 277 (13), 10912–10921. 10.1074/jbc.M111751200 11782487

[B20] Lee J. H. Kim C. Lee S. G. Yang W. M. Um J. Y. Sethi G. (2018). Ophiopogonin D modulates multiple oncogenic signaling pathways, leading to suppression of proliferation and chemosensitization of human lung cancer cells. Phytomedicine 40, 165–175. 10.1016/j.phymed.2018.01.002 29496169

[B21] Li W. Ji L. Tian J. Tang W. Shan X. Zhao P. (2021). Ophiopogonin D alleviates diabetic myocardial injuries by regulating mitochondrial dynamics. J. Ethnopharmacol. 271, 113853. 10.1016/j.jep.2021.113853 33485986

[B22] Liu Z. Liu G. Ha D. P. Wang J. Xiong M. Lee A. S. (2023). ER chaperone GRP78/BiP translocates to the nucleus under stress and acts as a transcriptional regulator. Proc. Natl. Acad. Sci. U. S. A. 120 (31), e2303448120. 10.1073/pnas.2303448120 37487081 PMC10400976

[B23] López-Gil J. C. García-Silva S. Ruiz-Cañas L. Navarro D. Palencia-Campos A. Giráldez-Trujillo A. (2024). The Peptidoglycan Recognition Protein 1 confers immune evasive properties on pancreatic cancer stem cells. Gut 73 (9), 1489–1508. 10.1136/gutjnl-2023-330995 38754953 PMC11347225

[B24] Lu Y. Huang R. Sun Z. Ou Y. (2023). A bovine milk-derived peptide ameliorates pancreatic β-cell dedifferentiation through PI3K/Akt/FOXO1 signaling in type 2 diabetes. Food Funct. 14 (17), 8018–8029. 10.1039/d3fo01330h 37593938

[B25] Luo D. Fan N. Zhang X. Ngo F. Y. Zhao J. Zhao W. (2022). Covalent inhibition of endoplasmic reticulum chaperone GRP78 disconnects the transduction of ER stress signals to inflammation and lipid accumulation in diet-induced obese mice. Elife 11, e72182. 10.7554/eLife.72182 35138251 PMC8828050

[B26] Nie Y. Li C. Sun N. (2023). Ophiopogonin D attenuates the progression of murine systemic lupus erythematosus by reducing B cell numbers. J. Biochem. Mol. Toxicol. 37 (7), e23361. 10.1002/jbt.23361 36999444

[B27] Nimmakayala R. K. Leon F. Rachagani S. Rauth S. Nallasamy P. Marimuthu S. (2021). Metabolic programming of distinct cancer stem cells promotes metastasis of pancreatic ductal adenocarcinoma. Oncogene 40 (1), 215–231. 10.1038/s41388-020-01518-2 33110235 PMC10041665

[B28] Qiao Y. Jiao H. Wang F. Niu H. (2020). Ophiopogonin D of Ophiopogon japonicus ameliorates renal function by suppressing oxidative stress and inflammatory response in streptozotocin-induced diabetic nephropathy rats. Braz J. Med. Biol. Res. 53 (7), e9628. 10.1590/1414-431x20209628 32520209 PMC7279694

[B29] Salinno C. Cota P. Bastidas-Ponce A. Tarquis-Medina M. Lickert H. Bakhti M. (2019). β-Cell maturation and identity in health and disease. Int. J. Mol. Sci. 20 (21), 5417. 10.3390/ijms20215417 31671683 PMC6861993

[B30] Shen X. Ruan Y. Zhao Y. Ye Q. Huang W. He L. (2024). Ophiopogonin D alleviates acute lung injury by regulating inflammation via the STAT3/A20/ASK1 axis. Phytomedicine 130, 155482. 10.1016/j.phymed.2024.155482 38824823

[B31] Shu W. Guo Z. Li L. Xiong Z. Wang Z. Yang Y. (2020). Regulation of molecular chaperone GRP78 by hepatitis B virus: control of viral replication and cell survival. Mol. Cell Biol. 40 (3), e00475. 10.1128/mcb.00475-19 31712392 PMC6965036

[B32] Siwan D. Nandave M. Gilhotra R. Almalki W. H. Gupta G. Gautam R. K. (2024). Unlocking β-cell restoration: the crucial role of PDX1 in diabetes therapy. Pathol. Res. Pract. 254, 155131. 10.1016/j.prp.2024.155131 38309018

[B33] Teo A. K. K. Lim C. S. Cheow L. F. Kin T. Shapiro J. A. Kang N. Y. (2018). Single-cell analyses of human islet cells reveal de-differentiation signatures. Cell Death Discov. 4, 14. 10.1038/s41420-017-0014-5 PMC5841351 29531811

[B34] Verfaillie T. Rubio N. Garg A. D. Bultynck G. Rizzuto R. Decuypere J. P. (2012). PERK is required at the ER-mitochondrial contact sites to convey apoptosis after ROS-based ER stress. Cell Death Differ. 19 (11), 1880–1891. 10.1038/cdd.2012.74 22705852 PMC3469056

[B35] Wang K. Cui X. Li F. Xia L. Wei T. Liu J. (2023). Glucagon receptor blockage inhibits β-cell dedifferentiation through FoxO1. Am. J. Physiol. Endocrinol. Metab. 324 (1), E97–e113. 10.1152/ajpendo.00101.2022 36383639

[B36] Wang L. Yang H. Qiao L. Liu J. Liao X. Huang H. (2022). Ophiopogonin D inhibiting epithelial NF-κB signaling pathway protects against experimental colitis in mice. Inflammation 45 (4), 1720–1731. 10.1007/s10753-022-01655-8 35460395

[B37] Wang T. Xia G. Li X. Gong M. Lv X. (2025). Endoplasmic reticulum stress in liver fibrosis: mechanisms and therapeutic potential. Biochim. Biophys. Acta Mol. Basis Dis. 1871 (3), 167695. 10.1016/j.bbadis.2025.167695 39864668

[B38] Weidemann B. J. Marcheva B. Kobayashi M. Omura C. Newman M. V. Kobayashi Y. (2024). Repression of latent NF-κB enhancers by PDX1 regulates β cell functional heterogeneity. Cell Metab. 36 (1), 90–102.e7. 10.1016/j.cmet.2023.11.018 38171340 PMC10793877

[B39] Yong J. Johnson J. D. Arvan P. Han J. Kaufman R. J. (2021). Therapeutic opportunities for pancreatic β-cell ER stress in diabetes mellitus. Nat. Rev. Endocrinol. 17 (8), 455–467. 10.1038/s41574-021-00510-4 34163039 PMC8765009

[B40] Zang Q. Q. Zhang L. Gao N. Huang C. (2016). Ophiopogonin D inhibits cell proliferation, causes cell cycle arrest at G2/M, and induces apoptosis in human breast carcinoma MCF-7 cells. J. Integr. Med. 14 (1), 51–59. 10.1016/s2095-4964(16)60238-8 26778229

[B41] Zhang H. Kang X. Ruan J. Ma L. Peng W. Shang H. (2023). Ophiopogonin D improves oxidative stress and mitochondrial dysfunction in pancreatic β cells induced by hydrogen peroxide through Keap1/Nrf2/ARE pathway in diabetes mellitus. Chin. J. Physiol. 66 (6), 494–502. 10.4103/cjop.CJOP-D-23-00069 38149562

[B43] Zhu Y. Liu Q. Zhou Z. Ikeda Y. (2017). PDX1, Neurogenin-3, and MAFA: critical transcription regulators for beta cell development and regeneration. Stem Cell Res. Ther. 8 (1), 240. 10.1186/s13287-017-0694-z 29096722 PMC5667467

