# Supplementary material for: Ophiopogonin D improves pancreatic islet cell dedifferentiation to treat diabetes by regulating the GRP78/ROS/PDX1 signaling pathway
Source: Front Pharmacol. 2025 Apr 29;16:1563201. doi: 10.3389/fphar.2025.1563201 (PMC12069403; doi:10.3389/fphar.2025.1563201)
Supplement: Supplementary file 1 [file DataSheet1.doc]

1. Detection of cell apoptosis by flow cytometryCells were processed according to the method described in section 2.11, collected, and stained using an apoptosis detection kit (Beyotime, Shanghai, China) following the manufacturer's instructions. The levels of cell apoptosis were observed using a flow cytometer. As shown in Figure S1, there was no significant change in the apoptosis levels of M group cells compared to the control group. The application of Op D also did not affect cell apoptosis. However, after the application of an endoplasmic reticulum stress inducer (TUN), the levels of cell apoptosis significantly increased, and Op D treatment could not reduce cell apoptosis levels.


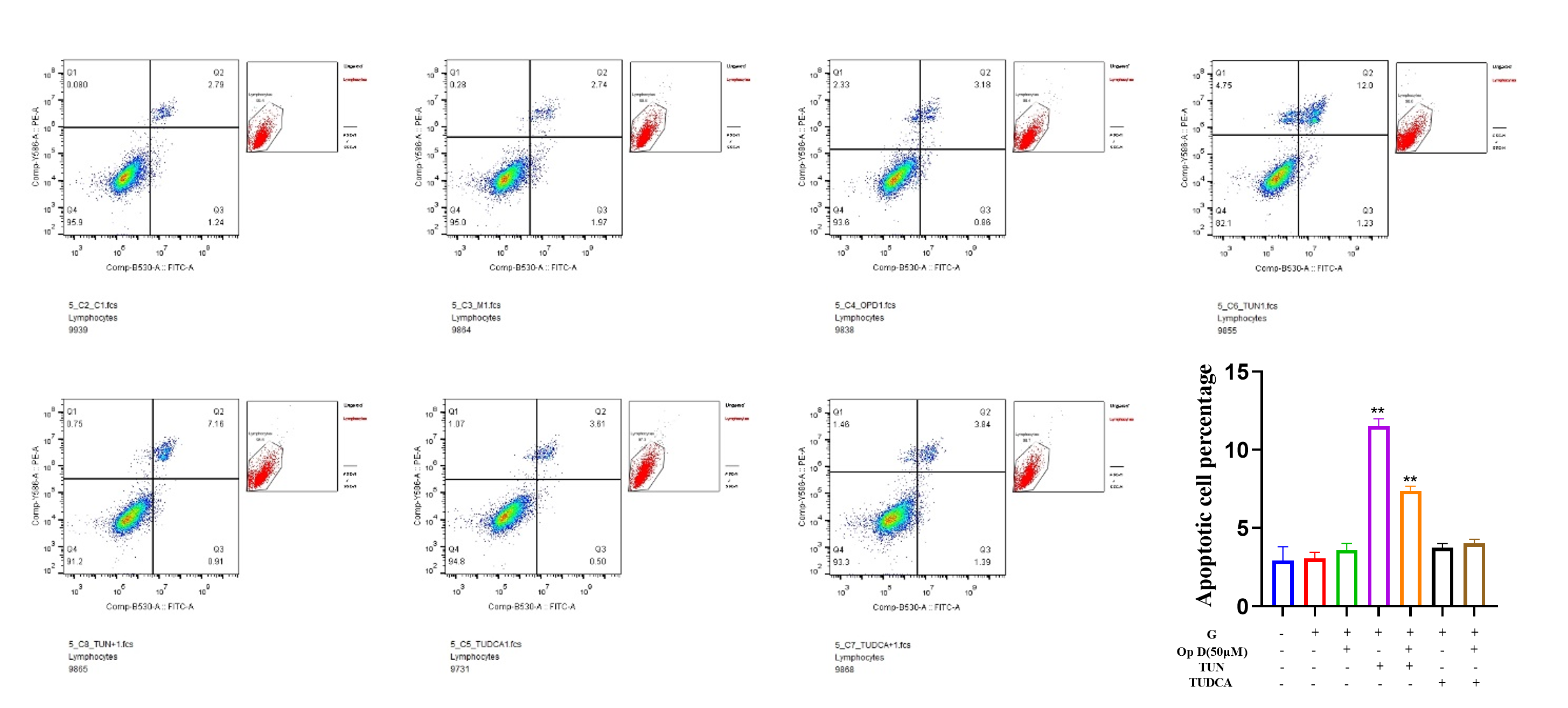


Figure S1 Cell apoptosis. ***P* < 0.01 versus M.
