# Supplementary material for: Ophiopogonin D improves pancreatic islet cell dedifferentiation to treat diabetes by regulating the GRP78/ROS/PDX1 signaling pathway
Source: Front Pharmacol. 2025 Apr 29;16:1563201. doi: 10.3389/fphar.2025.1563201 (PMC12069403; doi:10.3389/fphar.2025.1563201)
Supplement: Supplementary file 2 [file DataSheet2.doc]

1. Determination of Concentrations of Tunicamycin and Tauroursodeoxycholic Acid

To determine whether Op D improves the dedifferentiation of pancreatic β-cells by intervening in endoplasmic reticulum (ER) stress, we used the ER stress agonist tunicamycin and the ER stress inhibitor tauroursodeoxycholic acid (TUDCA) for further validation. Cells were seeded at a density of 8 × 10³ per well in a 96-well plate and treated with tunicamycin (TUN) at concentrations of 10 μM, 20 μM, 30 μM, 40 μM, 50 μM, 60 μM, 70 μM, and 80 μM, along with a control group. The TUDCA treatment groups included 10 μM, 20 μM, 30 μM, 40 μM, 50 μM, 60 μM, 70 μM, 80 μM, and 90 μM, also with a control group.

The results are shown in Figure S2. Based on our findings, we selected 10 μM TUN as a suitable concentration, noting that TUDCA was safe at concentrations below 90 μM. Consequently, we chose TUN concentrations of 2.5 μM, 5 μM, and 10 μM, and TUDCA concentrations of 20 μM, 40 μM, and 80 μM for further intervention on GRP78 expression levels. According to the results in Figure S2C, we decided to proceed with 5 μM TUN and 80 μM TUDCA for subsequent experiments.


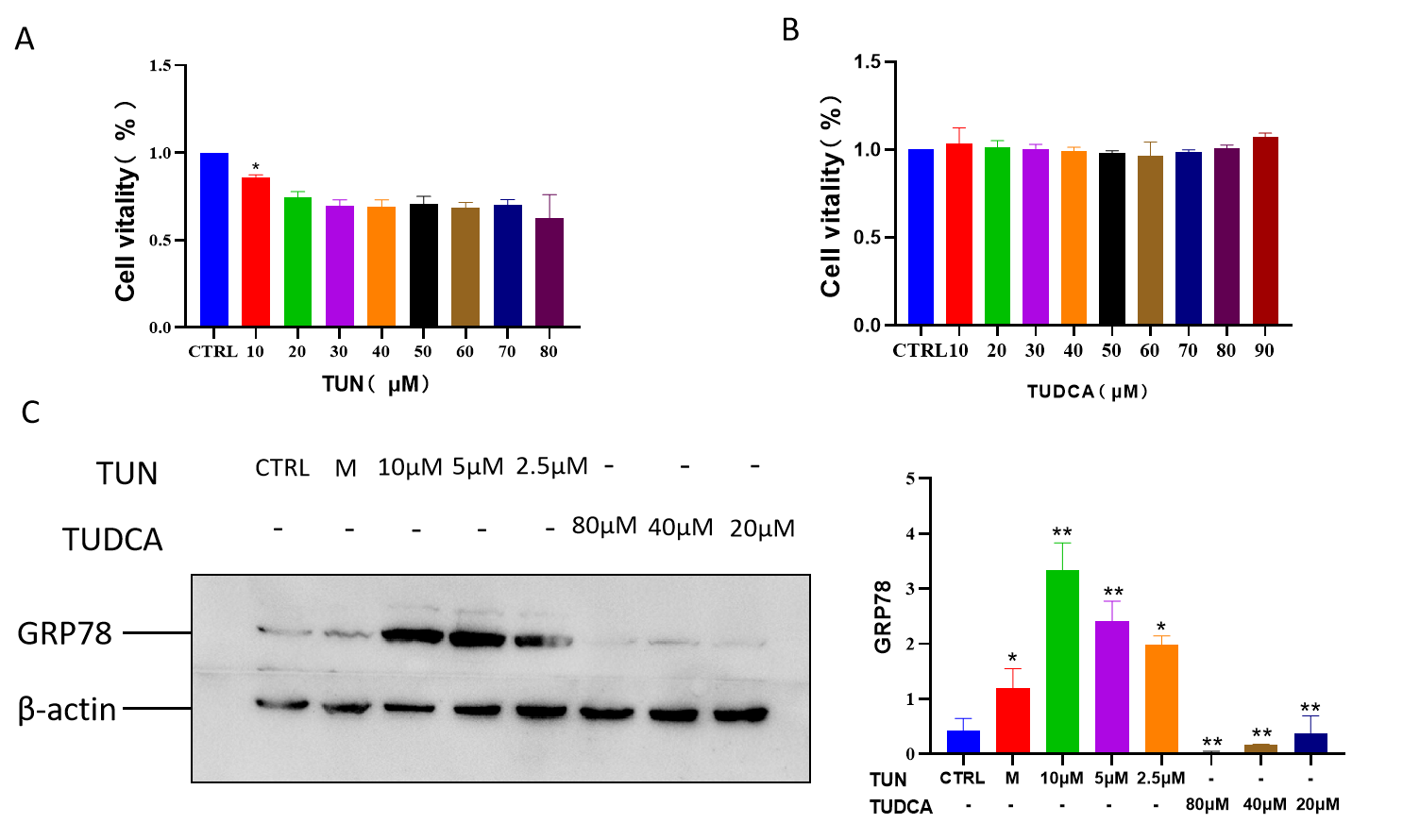


Figure S2 Selection of Concentrations for TUN and TUDCA. A. Exploration of safe concentrations for TUN;B. Exploration of safe concentrations for TUDCA;C. Effects of different concentrations of TUN and TUDCA on the expression levels of the endoplasmic reticulum stress-related protein GRP78. **P* < 0.05, ***P* < 0.01 versus M; ##*P* < 0.01 versus Ctrl.
